# Development of Novel Radiogallium-Labeled Bone Imaging Agents Using Oligo-Aspartic Acid Peptides as Carriers

**DOI:** 10.1371/journal.pone.0084335

**Published:** 2013-12-31

**Authors:** Kazuma Ogawa, Atsushi Ishizaki, Kenichiro Takai, Yoji Kitamura, Tatsuto Kiwada, Kazuhiro Shiba, Akira Odani

**Affiliations:** 1 Graduate School of Medical Sciences, Kanazawa University, Kanazawa, Japan; 2 Advanced Science Research Center, Kanazawa University, Kanazawa, Japan; University of Helsinki, Finland

## Abstract

^68^Ga (*T*_1/2_ = 68 min, a generator-produced nuclide) has great potential as a radionuclide for clinical positron emission tomography (PET). Because poly-glutamic and poly-aspartic acids have high affinity for hydroxyapatite, to develop new bone targeting ^68^Ga-labeled bone imaging agents for PET, we used 1,4,7,10-tetraazacyclododecane-1,4,7,10-tetraacetic acid (DOTA) as a chelating site and conjugated aspartic acid peptides of varying lengths. Subsequently, we compared Ga complexes, Ga-DOTA-(Asp)_n_ (n = 2, 5, 8, 11, or 14) with easy-to-handle ^67^Ga, with the previously described ^67^Ga-DOTA complex conjugated bisphosphonate, ^67^Ga-DOTA-Bn-SCN-HBP. After synthesizing DOTA-(Asp)_n_ by a Fmoc-based solid-phase method, complexes were formed with ^67^Ga, resulting in ^67^Ga-DOTA-(Asp)_n_ with a radiochemical purity of over 95% after HPLC purification. In hydroxyapatite binding assays, the binding rate of ^67^Ga-DOTA-(Asp)_n_ increased with the increase in the length of the conjugated aspartate peptide. Moreover, in biodistribution experiments, ^67^Ga-DOTA-(Asp)_8_, ^67^Ga-DOTA-(Asp)_11_, and ^67^Ga-DOTA-(Asp)_14_ showed high accumulation in bone (10.5±1.5, 15.1±2.6, and 12.8±1.7% ID/g, respectively) but were barely observed in other tissues at 60 min after injection. Although bone accumulation of ^67^Ga-DOTA-(Asp)_n_ was lower than that of ^67^Ga-DOTA-Bn-SCN-HBP, blood clearance of ^67^Ga-DOTA-(Asp)_n_ was more rapid. Accordingly, the bone/blood ratios of ^67^Ga-DOTA-(Asp)_11_ and ^67^Ga-DOTA-(Asp)_14_ were comparable with those of ^67^Ga-DOTA-Bn-SCN-HBP. In conclusion, these data provide useful insights into the drug design of ^68^Ga-PET tracers for the diagnosis of bone disorders, such as bone metastases.

## Introduction

Bone contains abundant proliferation factors, and is therefore a convenient environment for tumors to metastasize and grow. Indeed, malignant tumors frequently metastasize to the bone [1]. With the development of therapeutic methods and drugs, early diagnoses of bone metastases must be more important. Significant advances in imaging technologies such as X-ray computed tomography (CT) and magnetic resonance imaging (MRI) have been made during the last a few decades; however, because of its high sensitivity, nuclear medicine bone scanning is the optimal test for detecting bone metastases. Over the last thirty years, ^99m^Tc-bisphosphonate complexes such as methylenediphosphonate (^99m^Tc-MDP) and hydroxymethylenediphosphonate (^99m^Tc-HMDP) have been widely used as radiopharmaceuticals in bone scintigraphy for disorders such as metastatic bone cancer, Paget’s disease, and osteoporotic fractures [2]–[5]. The accumulation of ^99m^Tc-bisphoshonate complexes in bone must be derived from the binding of phosphonate groups in bisphosphonate to calcium (Ca^2+^) in hydroxyapatite crystals in bone, but the mechanism of high uptake to lesion sites has not been completely elucidated. One of factors should be the increased vascularity and regional blood flow caused from disease. However, it has been reported that regional bone blood flow alone does not account for the increased uptake of ^99m^Tc-bisphoshonate complexes [6]. Other factors should be involved in their binding and interaction with bone. It is generally assumed that ^99m^Tc-bisphoshonate complexes accumulate at sites of active bone metabolism, especially, at osteoblastic lesions [7], [8]. Newly formed bone has a much larger surface area than stable bone does. That is, the crystalline structure of hydroxyapatite in newly formed bone is amorphous and has a greater surface area than that in normal bone [9]. In the cases of ^99m^Tc-bisphoshonate complexes, the phosphonate groups coordinate with not only Ca^2+^ but also ^99m^Tc [10], which might decrease the inherent accumulation of bisphosphonate (MDP or HMDP) in bone. Incidentally, ^99m^Tc-bisphoshonate complexes cannot be isolated as well-defined single chemical species, but as mixtures of short- and long-chain oligomers, may reduce the efficacy of radiopharmaceuticals. Biological behaviors of these tracers are also affected by the degree of ionization and by variable oligomer constitutions of preparations [11]. To overcome the shortcomings of ^99m^Tc-bisphoshonate complexes, we and other groups have designed and developed ^99m^Tc-mononuclear complex-conjugated bisphosphonate compounds [12]–[15], in which phosphonate groups are not coordinated with ^99m^Tc. As expected, some of these compounds showed superior biodistribution compared with previous compounds. Of note, this drug concept is applicable to both ^99m^Tc-complex radiopharmaceuticals and other radiometals [16]–[26].

Sodium fluoride labeled with ^18^F (^18^F-NaF) for bone imaging was initially reported by Blau et al. in 1962 [27], and subsequently was approved by FDA in 1972. ^18^F-NaF accumulates in bone because fluoride anions are isomorphously exchanged with the hydroxyl group in hydroxyapatite (Ca_10_(PO_4_)_6_(OH)_2_) and fluoroapatite (Ca_10_(PO_4_)_6_F_2_) is formed. After the development of ^99m^Tc-labeled bone scintigraphy agents, such as ^99m^Tc-MDP, ^18^F-NaF was replaced by them because the physical characteristics of ^99m^Tc were more convenient for imaging with conventional gamma cameras in those days. However, in the last two decades, positron emission tomography (PET) and PET/CT have evolved significantly and become widespread. The changes caused the reemergence of ^18^F-NaF and bone imaging agents for PET are desired because current PET have higher spatial resolution and greater sensitivity than conventional gamma cameras. Actually, it was reported that ^18^F-NaF PET imaging was significantly more sensitive than ^99m^Tc-MDP planar and ^99m^Tc-MDP single photon emission computed tomography (SPECT) imaging [28]. However, most positron emitters, such as ^18^F, need high cost cyclotron facilities, and it limits the availability for PET.

Meanwhile, the radionuclide^ 68^Ga has great potential for clinical PET and could become an attractive alternative to ^18^F because of its radiophysical properties, particularly as a generator-produced nuclide with a half-life (*T*_1/2_) of 68 min [29]. Namely, it does not require an on-site cyclotron and can be eluted on demand. Indeed, in principle, the long half-life of the parent nuclide ^68^Ge (*T*_1/2_ = 270.8 days) provides a generator with a long life span. Therefore, the appearance of ^68^Ga-labeled compounds for bone imaging has been desired and some compounds have been reported in recent years [30]–[34].

Several noncollagenous bone proteins have repeating sequences of acidic amino acids (Asp or Glu) in their structures, offering potential hydroxyapatite-binding sites. For example, osteopontin and bone sialoprotein, 2 major noncollagenous bone matrix proteins, have repeating Asp and Glu rich sequences, respectively [35]–[37]. Reportedly, poly-glutamic and poly-aspartic acids have high affinity for hydroxyapatite and could be used to deliver drugs to bone tissues [38]–[40].

**Figure 1:**
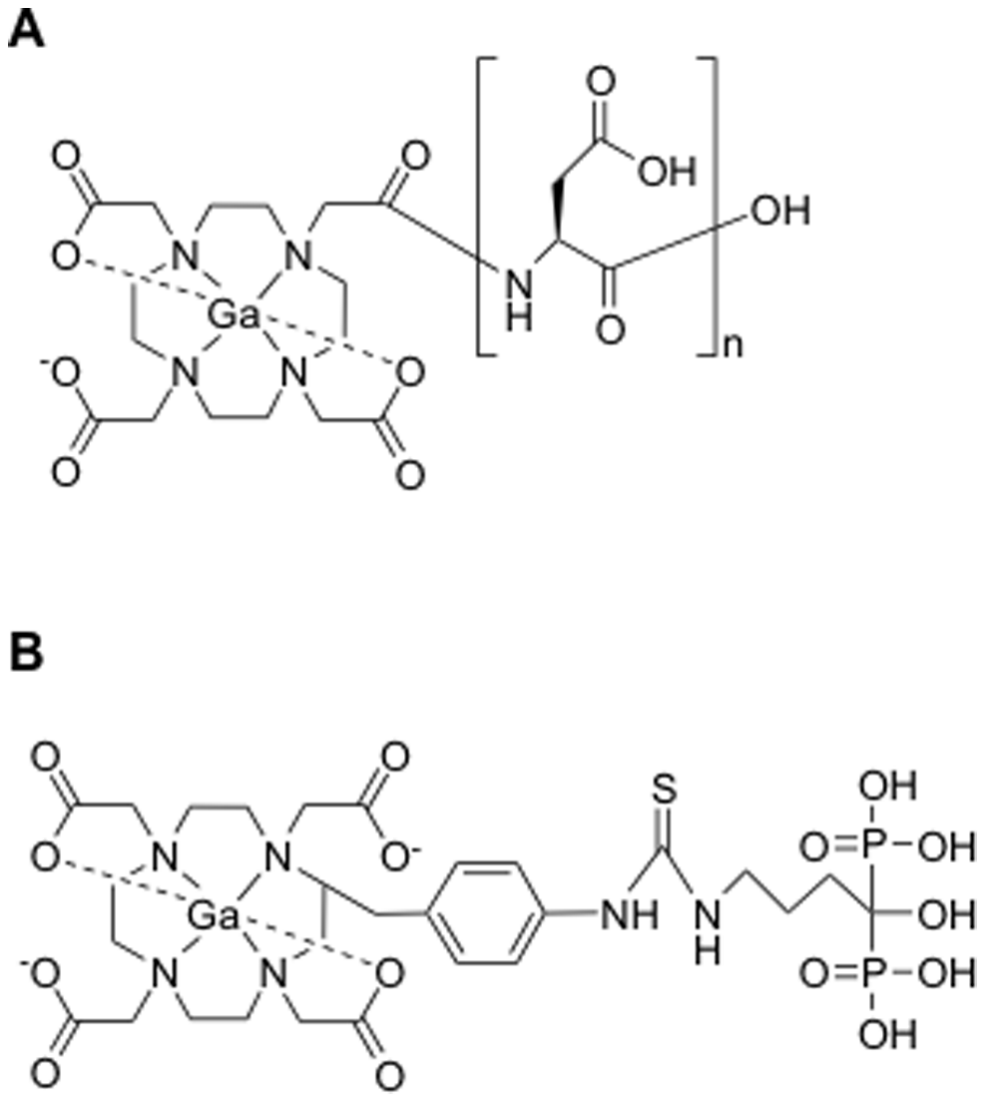
Structures. Chemical structures of (A) Ga-DOTA-(Asp)_n_ (n = 2, 5, 8, 11, or 14) and (B) Ga-DOTA-Bn-SCN-HBP.

In this study, to develop new PET tracers for imaging bone disorders such as bone metastases, because it is well known that ^68^Ga forms a stable complex with 1,4,7,10-tetraazacyclododecane-1,4,7,10-tetraacetic acid (DOTA), DOTA was chosen as chelating sites. Subsequently, a series of Ga-DOTA-conjugated acidic amino acid peptides (Ga-DOTA-(Asp)_n_; Figure 1A) of varying peptide lengths (n = 2, 5, 8, 11, or 14) were designed using the easy-to-handle radioisotope ^67^Ga, and these were evaluated and compared, *in vitro* and *in vivo*, with the previously developed conjugated bisphosphonate complex ^67^Ga-DOTA-Bn-SCN-HBP (Figure 1B) [33].

## Materials and Methods

### Materials

Electrospray ionization mass spectra (ESI-MS) were obtained with a LCQ (Thermo Fisher Scientific, Waltham, MA, USA). Matrix assisted laser desorption/ionization-time of flight mass spectra (MALDI-TOF-MS) were obtained with ABI 4800 plus (AB SCIEX, Foster, CA, USA). [^67^Ga]GaCl_3_ was supplied by Nihon Medi-Physics Co., Ltd. (Tokyo, Japan). 1,4,7,10-Tetraazacyclododecane-1,4,7-tris(t-butyl acetate) (DOTA-tris) was purchased from Macrocyclics (Dallas, TX, USA). 9-Fluorenylmethoxycarbonyl (Fmoc)-Asp(OtBu)-Wang resin and Fmoc-Asp(OtBu) were purchased from Merck KGaA (Darmstadt, Germany). Alendronate was synthesized according to the previous reported method [18]. Other reagents were of reagent grade and used as received.

### Synthesis of DOTA-(Asp)_n_

The protected peptidyl resin was manually constructed by an Fmoc-based solid-phase methodology using Fmoc-Asp(OtBu)-Wang resin and Fmoc-Asp(OtBu). The peptide chain was constructed in cycles of (I) 15 minutes of deprotection with 20% piperidine in dimethylformamide (DMF) and (II) 2 hours of coupling with 3 equivalents of Fmoc-Asp(OtBu), 1,3-diisopropylcarbodiimide (DIPCDI) and 1-hydroxybenzotriazole hydrate (HOBt) in DMF. The coupling reaction was then repeated after Kaiser test was positive for the resin [41]. After construction of the peptide chain on the resin, the Fmoc protecting group was removed using 20% piperidine in DMF, and a mixture containing 2 equivalents of DOTA-tris, DIPCDI, and HOBt in DMF was added and allowed to react for 2 hours, as described above. To cleave peptides from the resin and deprotect, 0.5 mL of thioanisole and 5 mL of trifluoroacetic acid (TFA) were added to the fully protected peptide resin at 0°C and stirred at room temperature for 2 hours. After resin removal by filtration, ether was added to the filtrate at 0°C to precipitate crude peptide. The crude products were purified by reversed-phase (RP)-HPLC performed with a Hydrosphere 5C18 column (10×150 mm; YMC, Kyoto, Japan) at a flow rate of 4 mL/min with an isocratic mobile phase of water containing 0.1% TFA [in the case of DOTA-(Asp)_2_] or with a Cosmosil 5C_18_-AR 300 column (10×150 mm; Nacalai Tesque, Kyoto, Japan) at a flow rate of 4 mL/min with a 0–20% methanol gradient mobile phase of 0.1% TFA in water over 20 minutes [in the case of DOTA-(Asp)_n_ (n = 5, 8, 11, or 14)], respectively. Chromatograms were obtained by monitoring the UV adsorption at a wavelength of 220 nm. The fraction containing DOTA-(Asp)_n_ (n = 2, 5, 8, 11, or 14) was determined by mass spectrometry, and collected. The solvent was removed by lyophilization to provide DOTA-(Asp)_n_ as white powder.

DOTA-(Asp)_2_ MS (ESI): *m/z* 635 (M+H)^+^, Yield : 22.0%

DOTA-(Asp)_5_ MS (ESI): *m/z* 980 (M+H)^+^, Yield : 18.6%

DOTA-(Asp)_8_ MS (ESI): *m/z* 1325 (M+H)^+^, Yield : 36.0%

DOTA-(Asp)_11_ MS (ESI): *m/z* 1670 (M+H)^+^, Yield : 20.2%

DOTA-(Asp)_14_ MS (MALDI): *m/z* 2015 (M+H)^+^, Yield : 5.0%

### Preparation of Ga-DOTA-(Asp)_n_ (n = 2, 5, 8, 11, or 14)

DOTA-(Asp)_n_ (n = 2, 5, 8, 11, or 14) (1 µmol) was dissolved in 75 µL of water, and Ga(NO_3_)_3_ (0.77 mg, 3 µmol) was added to the DOTA-(Asp)_n_ solution. The mixture was reacted at 40°C for 2 hours. Ga-DOTA-(Asp)_n_ was purified by RP-HPLC performed with a Hydrosphere 5C18 column (4.6×250 mm; YMC) at a flow rate of 1 mL/min with an isocratic mobile phase of water containing 0.1% TFA [in the case of Ga-DOTA-(Asp)_2_] or with a Cosmosil 5C_18_-AR 300 column (4.6×150 mm) at a flow rate of 1 mL/min with a 0–20% methanol gradient mobile phase of 0.1% TFA in water over 20 minutes [in the case of DOTA-(Asp)_n_ (n = 5, 8, 11, or 14)]. Chromatograms were obtained by monitoring the UV adsorption at a wavelength of 220 nm. The fraction containing DOTA-(Asp)_n_ (n = 2, 5, 8, 11, or 14) was determined by mass spectrometry, and collected.

Ga-DOTA-(Asp)_2_ MS (ESI): *m/z* 701 (M+H)^+^

Ga-DOTA-(Asp)_5_ MS (ESI): *m/z* 1046 (M+H)^+^

Ga-DOTA-(Asp)_8_ MS (ESI): *m/z* 1391 (M+H)^+^

Ga-DOTA-(Asp)_11_ MS (ESI): *m/z* 1736 (M+H)^+^

Ga-DOTA-(Asp)_14_ MS (MALDI): *m/z* 2081 (M+H)^+^

### Preparation of ^67^Ga-DOTA-(Asp)_n_ (n = 2, 5, 8, 11, or 14)

Approximately 50 µg of DOTA-(Asp)_n_ (n = 2, 5, 8, 11, or 14) conjugates were dissolved in 75 µL of 0.2 M ammonium acetate buffer (pH 5.0), and 25 µL of ^67^GaCl_3_ solution (1.85 MBq) in 0.01 M HCl was added and allowed to react at 80°C for 8 minutes. ^67^Ga-DOTA-(Asp)_n_ was purified by RP-HPLC under the conditions described for Ga-DOTA-(Asp)_n_.

### Hydroxyapatite-binding Assays

Hydroxyapatite-binding assays were performed according to previously described procedures with slight modifications [33]. In brief, hydroxyapatite beads (Bio-Gel; Bio-Rad, Hercules, CA, USA) were suspended in Tris/HCl-buffered saline (50 mM, pH 7.4) at 2.5 mg/mL, 10 mg/mL, and 25 mg/mL. For the solutions of ^67^Ga-DOTA-(Asp)_n_ (n = 2, 5, 8, 11, or 14), the ligand concentrations were adjusted to 19.5 µM by adding DOTA-(Asp)_n_. Two hundred microliters of each ^67^Ga-DOTA-(Asp)_n_ solution was added to 200 µL of the hydroxyapatite suspension, and the samples were gently shaken for 1 hour at room temperature. After centrifugation at 10,000 *g* for 5 minutes, the radioactivity of the supernatants was measured using an auto well gamma counter (ARC-7010B, Hitachi Aloka Medical, Ltd., Tokyo, Japan). Control experiments were performed using the same procedure without hydroxyapatite beads, which showed less than 0.1% adsorption of radioactivity to vials. The ratios of binding were determined as follows:
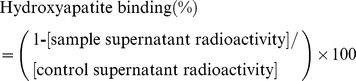



The effect of bisphosphonate on the binding of ^67^Ga-DOTA-(Asp)_14_ or ^67^Ga-DOTA-Bn-SCN-HBP to hydroxyapatite beads was also examined. In these experiments, 100 µL of ^67^Ga-DOTA-(Asp)_14_ or ^67^Ga-DOTA-Bn-SCN-HBP solutions containing varying concentrations of the bisphosphonate compound alendronate were incubated with 100 µL of suspensions containing 1 mg of hydroxyapatite beads. After centrifugation, the radioactivity of the supernatant was measured, and hydroxyapatite-binding ratios were calculated as described above.

### Biodistribution Experiments

Experiments with animals were conducted in strict accordance with the Guidelines for the Care and Use of Laboratory Animals of Kanazawa University. The animal experimental protocols used were approved by the Committee on Animal Experimentation of Kanazawa University (Permit Number: AP-132633). Biodistribution experiments were performed after an intravenous administration of each diluted tracer solution (37 kBq/100 µL) to 6-week-old male ddY mice (27–32 g, Japan SLC, Inc., Hamamatsu, Japan). To investigate the effect of an excess amount of bisphosphonate on biodistribution, alendronate (20 mg/kg) was intravenously administered to mice 1 minute before the intravenous injection of ^67^Ga-DOTA-(Asp)_14_ or ^67^Ga-DOTA-Bn-SCN-HBP. Four to six mice each were sacrificed by decapitation at 10, 60, and 180 minutes post-injection. Tissues of interest were removed and weighed. Complete left femurs were isolated as representative bone samples, radioactivity was determined using an auto well gamma counter, and counts were corrected for background radiation and physical decay during counting.

### Protein-binding Assay

Serum protein binding ratios of ^67^Ga-DOTA-(Asp)_n_ (n = 2, 5, 8, 11, or 14) and ^67^Ga-DOTA-Bn-SCN-HBP were evaluated by an ultrafiltration method. In these experiments, 6-week-old male ddY mice received intravenous boluses of radiotracer. After 3 minutes, the mice were anesthetized with ether, and blood was collected by heart puncture. Serum samples were prepared and applied to an Amicon Ultra-0.5 Centrifugal Filter Unit with Ultracel-30 membrane (Millipore). The units were centrifuged at 14,000 *g* for 20 minutes at room temperature. The radioactivity counts of the initials and filtrates were determined using an auto well gamma counter. The protein-binding ratios were then calculated as follows:
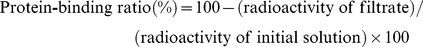



### Statistical Analysis

Data are expressed as means ± standard deviations where appropriate. In biodistribution experiments using alendronate as a blocking agent, differences were identified using unpaired Students’ *t* test and were considered significant when *p*<0.05.

## Results

### Preparation of ^67^Ga-DOTA-(Asp)_n_ (n = 2, 5, 8, 11, or 14)

**Figure 2:**
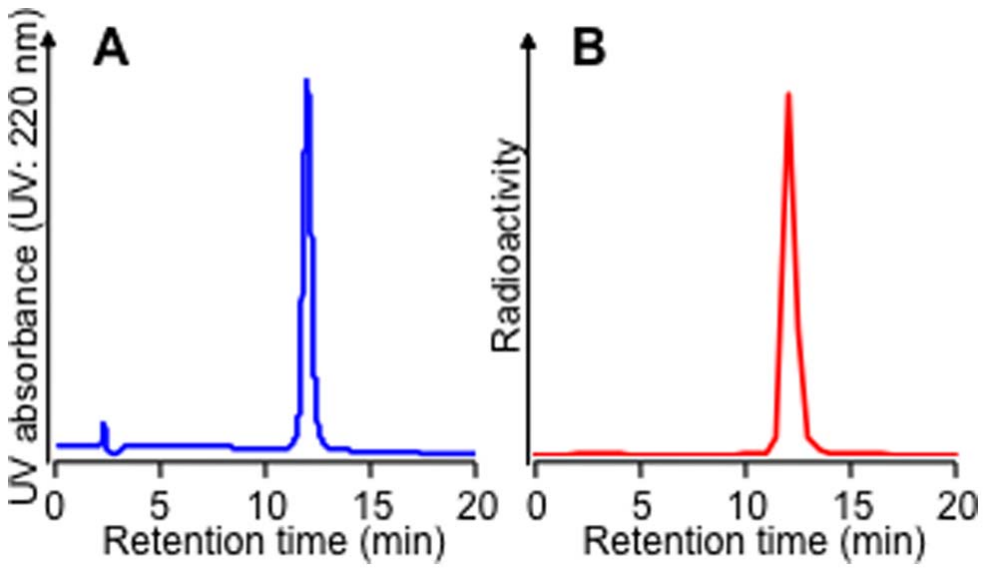
RP-HPLC chromatograms. RP-HPLC chromatograms of (A) nonradioactive Ga-DOTA-(Asp)_14_ and (B) ^67^Ga-DOTA-(Asp)_14_. Conditions: A flow rate of 1 mL/min with a gradient mobile phase of 100% water containing 0.1% TFA to 20% methanol in water containing 0.1% TFA for 20 minutes.

^67^Ga-DOTA-(Asp)_n_ (n = 2, 5, 8, 11, or 14) was prepared by complexation of DOTA-(Asp)_n_ with ^67^Ga. Radiochemical yields of ^67^Ga-DOTA-(Asp)_2_, ^67^Ga-DOTA-(Asp)_5_, ^67^Ga-DOTA-(Asp)_8_,^ 67^Ga-DOTA-(Asp)_11_, and^ 67^Ga-DOTA-(Asp)_14_, were 25%, 67%, 74%, 56%, and 51%, respectively. After RP-HPLC purification, ^67^Ga-DOTA-(Asp)_n_ had a radiochemical purity of over 95%. Figure 2 shows typical chromatograms of ^67^Ga-DOTA-(Asp)_14_ and Ga-DOTA-(Asp)_14_. RP-HPLC analyses of ^67^Ga-DOTA-(Asp)_n_ and Ga-DOTA-(Asp)_n_ showed similar retention times, indicating that the radiogallium-labeled product was identical to its authentic nonradioactive counterpart.

### Hydroxyapatite-binding Assay

**Figure 3:**
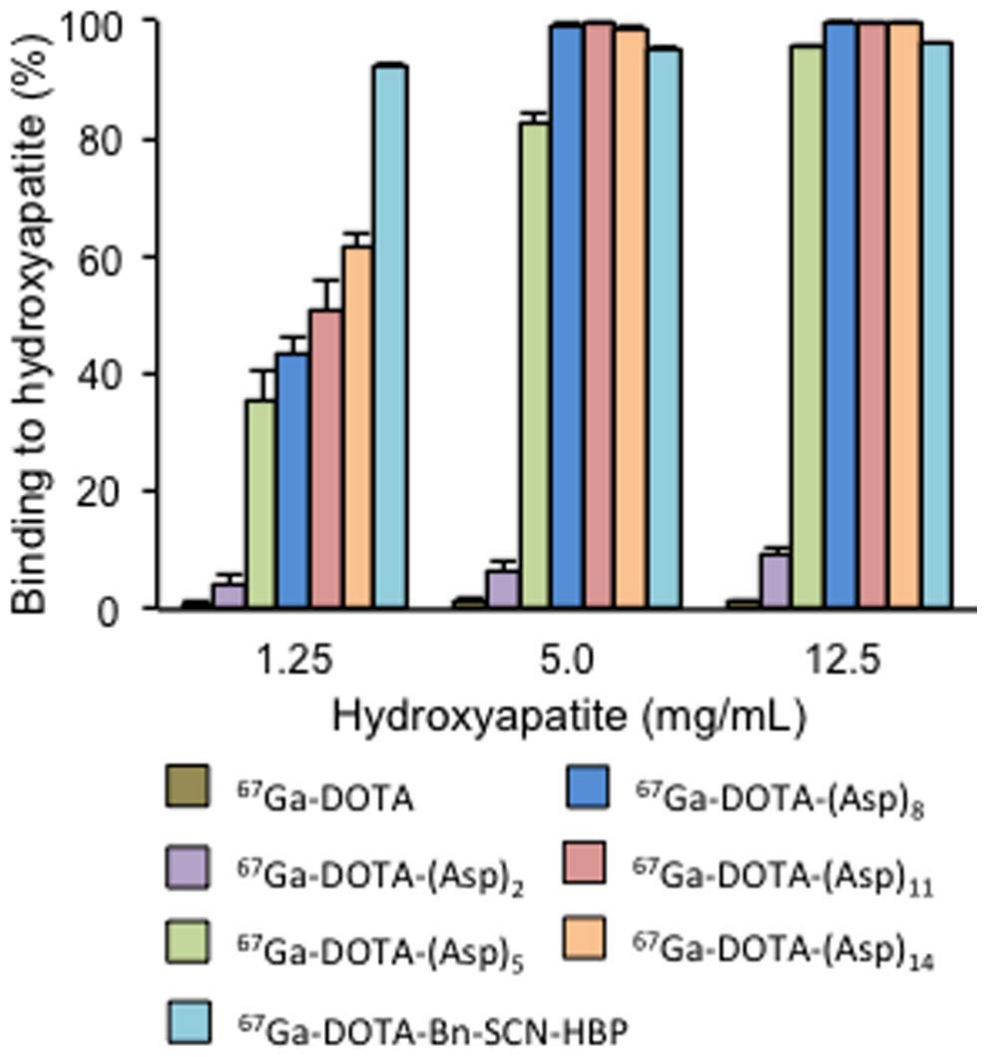
Hydroxyapatite binding assay. Binding ratios of ^67^Ga-DOTA, ^67^Ga-DOTA-(Asp)_n_ (n = 2, 5, 8, 11, or 14), and ^67^Ga-DOTA-Bn-SCN-HBP to hydroxyapatite beads. Data are expressed as the mean ± SD for four samples.

**Figure 4:**
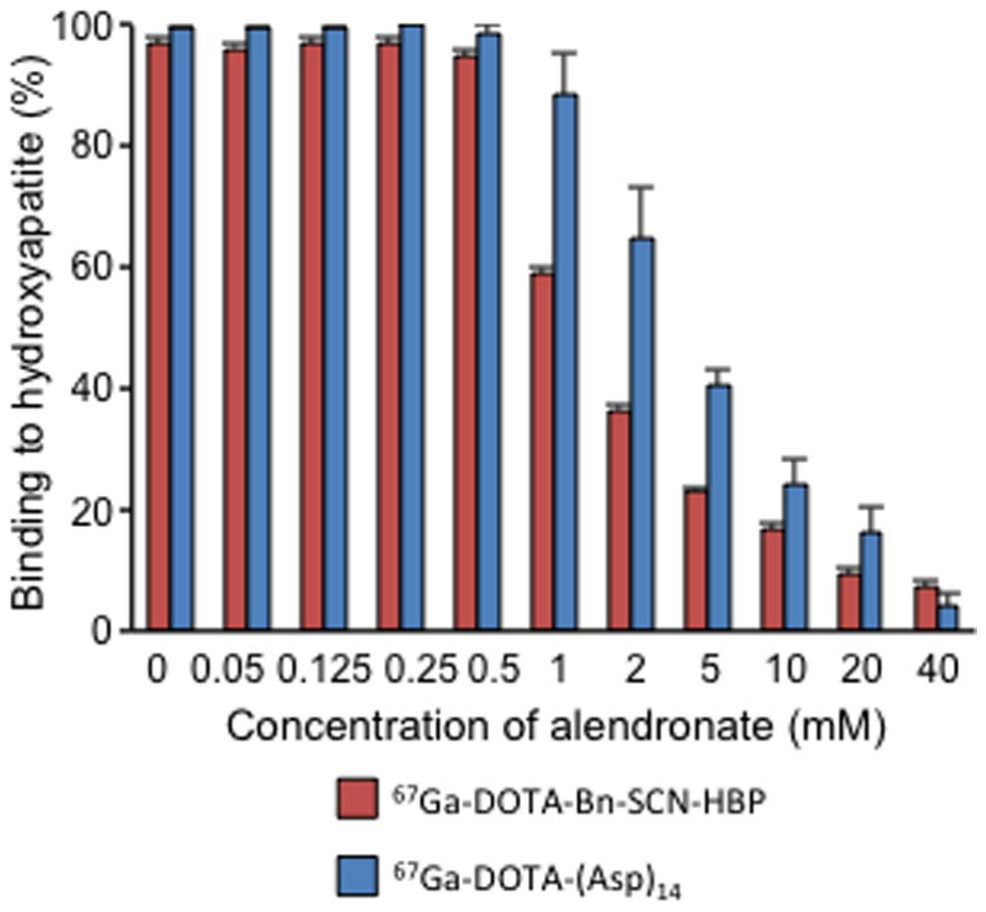
Inhibition using alendronate. Binding ratios of ^67^Ga-DOTA-(Asp)_14_ and ^67^Ga-DOTA-Bn-SCN-HBP to hydroxyapatite in the presence of various concentrations of the inhibitor alendronate. Data are expressed as the mean ± SD for four samples.

Figure 3 shows the percentage of each ^67^Ga-DOTA-(Asp)_n_ (n = 2, 5, 8, 11, or 14) that was bound to hydroxyapatite beads. Binding of each ^67^Ga-DOTA-(Asp)_n_ to hydroxyapatite beads increased with the amount of hydroxyapetite. In contrast, ^67^Ga-DOTA and ^67^Ga-DOTA-(Asp)_2_ hardly bound to hydroxyapatite beads. Moreover, hydroxyapetite binding of ^67^Ga-DOTA-(Asp)_n_ increased with the increase in the length of the aspartic acid chain. On the other hand, the binding of ^67^Ga-DOTA-(Asp)_14_ and ^67^Ga-DOTA-Bn-SCN-HBP was inhibited by the addition of a bisphosphonate compound alendronate in a concentration-dependent manner (Figure 4). Although ^67^Ga-DOTA-Bn-SCN-HBP had higher affinity for hydroxyapatite than ^67^Ga-DOTA-(Asp)_14_ (Figure 3), ^67^Ga-DOTA-Bn-SCN-HBP appeared more susceptible to alendronate inhibition than ^67^Ga-DOTA-(Asp)_14_ (Figure 4).

### Biodistribution Experiments

The biodistributions of ^67^Ga-DOTA-(Asp)_n_ compounds (n = 2, 5, 8, 11, or 14) in normal mice are listed in Tables 1–5. Among these, ^67^Ga-DOTA-(Asp)_8_, ^67^Ga-DOTA-(Asp)_11_, and ^67^Ga-DOTA-(Asp)_14_ showed higher accumulation, and led to sustained radioactivity in the femur. Though ^67^Ga-DOTA-(Asp)_5_ led to moderate accumulation of radioactivity in the femur at 10 min after injection, the radioactivity was not retained. Meanwhile, ^67^Ga-DOTA-(Asp)_2_ hardly accumulated in the femur, and almost all injected radioactivity was rapidly excreted via the kidneys. Almost all radioactivity except radioactivity in bone after injection of ^67^Ga-DOTA-(Asp)_n_ compounds (n = 5, 8, 11, or 14) was also quickly excreted via the kidneys. Consequently, radioactivity was scarcely observed in any tissues except the bone and kidney at 60 minutes after injection of ^67^Ga-DOTA-(Asp)_n_ compounds (n = 5, 8, 11, or 14).

**Table 1:** Biodistribution of radioactivity after intravenous administration of ^67^Ga-DOTA-(Asp)_2_ in mice.^a^

	Time after administration		
Tissue	10 min	60 min	180 min
Blood	2.69 (0.42)	0.24 (0.08)	0.07 (0.06)
Liver	0.67 (0.11)	0.31 (0.28)	0.11 (0.02)
Kidney	11.77 (1.92)	8.87 (4.09)	1.31 (0.50)
Small-intestine	0.57 (0.08)	0.22 (0.12)	0.08 (0.03)
Large-intestine	0.46 (0.07)	0.07 (0.01)	0.40 (0.30)
Spleen	0.61 (0.01)	0.14 (0.04)	0.12 (0.02)
Pancreas	0.76 (0.14)	0.16 (0.05)	0.08 (0.03)
Lung	2.10 (0.22)	0.24 (0.06)	0.08 (0.02)
Heart	0.98 (0.15)	0.13 (0.05)	0.06 (0.04)
Stomach^b^	0.28 (0.05)	0.07 (0.04)	0.05 (0.04)
Bone (Femur)	1.48 (0.31)	0.80 (0.40)	0.38 (0.15)
Muscle	0.76 (0.28)	0.13 (0.05)	0.07 (0.02)
Brain	0.08 (0.02)	0.02 (0.01)	0.01 (0.01)
F/B ratio^c^	0.55 (0.07)	3.28 (1.23)	8.04 (4.50)

^a^ Expressed as % injected dose. Each value represents the mean (SD) for five animals.

^b^ Expressed as % injected dose.

^c^ Femur:blood ratio.

**Table 2:** Biodistribution of radioactivity after intravenous administration of ^67^Ga-DOTA-(Asp)_5_ in mice.^a^

	Time after administration		
Tissue	10 min	60 min	180 min
Blood	2.18 (0.13)	0.16 (0.04)	0.02 (0.01)
Liver	0.44 (0.07)	0.07 (0.01)	0.04 (0.01)
Kidney	8.42 (1.82)	3.61 (1.12)	0.91 (0.27)
Small-intestine	0.42 (0.06)	0.11 (0.04)	0.03 (0.01)
Large-intestine	0.37 (0.04)	0.19 (0.15)	0.07 (0.01)
Spleen	0.50 (0.04)	0.07 (0.02)	0.03 (0.00)
Pancreas	0.65 (0.07)	0.13 (0.07)	0.02 (0.00)
Lung	1.50 (0.13)	0.15 (0.04)	0.02 (0.01)
Heart	0.79 (0.08)	0.07 (0.02)	0.03 (0.01)
Stomach^b^	0.24 (0.03)	0.05 (0.02)	0.03 (0.01)
Bone (Femur)	6.36 (0.86)	3.63 (0.29)	1.76 (0.04)
Muscle	0.63 (0.21)	0.09 (0.05)	0.03 (0.01)
Brain	0.08 (0.02)	0.01 (0.00)	0.00 (0.00)
F/B ratio^c^	2.91 (0.34)	23.33 (4.84)	85.54 (26.61)

^a^ Expressed as % injected dose. Each value represents the mean (SD) for four animals.

^b^ Expressed as % injected dose.

^c^ Femur:blood ratio.

**Table 3:** Biodistribution of radioactivity after intravenous administration of ^67^Ga-DOTA-(Asp)_8_ in mice.^a^

	Time after administration		
Tissue	10 min	60 min	180 min
Blood	2.06 (0.18)	0.14 (0.07)	0.07 (0.01)
Liver	0.46 (0.06)	0.10 (0.08)	0.07 (0.01)
Kidney	9.88 (5.74)	2.60 (2.40)	1.20 (0.61)
Small-intestine	0.49 (0.05)	0.15 (0.10)	0.09 (0.03)
Large-intestine	0.39 (0.06)	0.05 (0.01)	0.17 (0.07)
Spleen	0.42 (0.09)	0.11 (0.15)	0.06 (0.01)
Pancreas	0.56 (0.14)	0.07 (0.04)	0.05 (0.02)
Lung	1.59 (0.16)	0.14 (0.05)	0.07 (0.02)
Heart	1.09 (0.44)	0.06 (0.02)	0.04 (0.03)
Stomach^b^	0.29 (0.16)	0.05 (0.05)	0.04 (0.03)
Bone (Femur)	11.65 (0.49)	12.56 (3.09)	11.29 (0.62)
Muscle	0.87 (0.51)	0.09 (0.06)	0.09 (0.07)
Brain	0.10 (0.04)	0.03 (0.02)	0.02 (0.02)
F/B ratio^c^	5.69 (0.42)	102.00 (41.75)	171.85 (26.47)

^a^ Expressed as % injected dose. Each value represents the mean (SD) for five animals.

^b^ Expressed as % injected dose.

^c^ Femur:blood ratio.

**Table 4:** Biodistribution of radioactivity after intravenous administration of ^67^Ga-DOTA-(Asp)_11_ in mice.^a^

	Time after administration		
Tissue	10 min	60 min	180 min
Blood	1.51 (0.16)	0.11 (0.02)	0.03 (0.01)
Liver	0.44 (0.12)	0.07 (0.01)	0.05 (0.01)
Kidney	16.04 (7.49)	1.76 (1.38)	0.80 (0.21)
Small-intestine	0.41 (0.04)	0.27 (0.37)	0.05 (0.02)
Large-intestine	0.27 (0.02)	0.06 (0.06)	0.13 (0.05)
Spleen	0.31 (0.08)	0.09 (0.06)	0.04(0.02)
Pancreas	0.62 (0.20)	0.07 (0.06)	0.05 (0.01)
Lung	1.11 (0.16)	0.12 (0.09)	0.04 (0.01)
Heart	0.55 (0.04)	0.08 (0.05)	0.02 (0.02)
Stomach^b^	0.21 (0.02)	0.18 (0.35)	0.04 (0.01)
Bone (Femur)	13.09 (1.16)	16.30 (3.58)	13.91 (1.93)
Muscle	0.48 (0.06)	0.13 (0.11)	0.08 (0.05)
Brain	0.05 (0.02)	0.02 (0.01)	0.01 (0.01)
F/B ratio^c^	8.71 (0.73)	156.07 (45.00)	501.32 (156.89)

^a^ Expressed as % injected dose. Each value represents the mean (SD) for five animals.

^b^ Expressed as % injected dose.

^c^ Femur:blood ratio.

**Table 5:** Biodistribution of radioactivity after intravenous administration of ^67^Ga-DOTA-(Asp)_14_ in mice.^a^

	Time after administration		
Tissue	10 min	60 min	180 min
Blood	1.61 (0.09)	0.07 (0.01)	0.03 (0.01)
Liver	0.39 (0.06)	0.05 (0.01)	0.05 (0.01)
Kidney	12.43 (5.81)	0.99 (0.17)	0.76 (0.10)
Small-intestine	0.38 (0.06)	0.08 (0.02)	0.05 (0.02)
Large-intestine	0.30 (0.04)	0.04 (0.01)	0.15 (0.08)
Spleen	0.42 (0.11)	0.07 (0.02)	0.03 (0.01)
Pancreas	0.50 (0.02)	0.07 (0.02)	0.03 (0.01)
Lung	1.19 (0.14)	0.08 (0.01)	0.03 (0.00)
Heart	0.62 (0.06)	0.06 (0.00)	0.03 (0.00)
Stomach^b^	0.24 (0.06)	0.10 (0.07)	0.02 (0.01)
Bone (Femur)	10.08 (0.86)	12.81 (1.67)	13.27 (1.15)
Muscle	0.57 (0.19)	0.08 (0.02)	0.05 (0.03)
Brain	0.05 (0.01)	0.01 (0.00)	0.01 (0.00)
F/B ratio^c^	6.27 (0.66)	185.54 (46.89)	591.08 (221.41)

^a^ Expressed as % injected dose. Each value represents the mean (SD) for five animals.

^b^ Expressed as % injected dose.

^c^ Femur:blood ratio.

The biodistribution of ^67^Ga-DOTA-(Asp)_14_ after pretreatment of normal mice with alendronate (20 mg/kg) is presented in Table 6. Table 7 and reference 29 show biodistribution of ^67^Ga-DOTA-Bn-SCN-HBP with or without pretreatment of alendronate (20 mg/kg). Although pretreatment with the same dose of alendronate significantly inhibited the accumulation of ^67^Ga-DOTA-Bn-SCN-HBP in bone (17.44±1.12 and 8.59±0.81%ID/g at 10 min, 22.28±2.15 and 13.47±1.76%ID/g at 1 h, 23.53±2.34 and 13.30±2.10%ID/g at 3 h), alendronate had comparatively little effect on the bone accumulation of ^67^Ga-DOTA-(Asp)_14_.

**Table 6:** Biodistribution of radioactivity after intravenous administration of ^67^Ga-DOTA-(Asp)_14_ in mice with pretreatment of alendronate (20 mg/kg).^a^

	Time after administration		
Tissue	10 min	60 min	180 min
Blood	1.94 (0.33)	0.23 (0.05) **	0.06 (0.02) **
Liver	2.20 (0.50)**	1.39 (0.29)**	1.27 (0.72)**
Kidney	16.66 (5.78)	3.28 (0.82)**	3.34 (0.92)**
Small-intestine	0.53 (0.09)*	0.22 (0.05)**	0.29 (0.12)**
Large-intestine	0.40 (0.08)*	0.08 (0.01)**	0.33 (0.07)**
Spleen	1.86 (0.98)*	1.03 (0.35)**	0.89 (0.59)*
Pancreas	0.72 (0.12)**	0.15 (0.02)**	0.12 (0.07)*
Lung	5.86 (3.64)*	4.07 (2.99)*	2.49 (1.78)*
Heart	0.88 (0.18)*	0.24 (0.08)**	0.15 (0.07)**
Stomach^b^	0.36 (0.09)*	0.15 (0.12)	0.11 (0.04)**
Bone (Femur)	8.59 (0.55)**	11.81 (1.95)	12.88 (2.30)
Muscle	0.53 (0.11)	0.11 (0.07)	0.05 (0.02)
Brain	0.05 (0.01)	0.02 (0.00) *	0.01 (0.00)
F/B ratio^c^	4.50 (0.63)**	52.96 (17.28)**	229.73 (67.08)**

^a^ Expressed as % injected dose. Each value represents the mean (SD) for five or six animals.

^b^ Expressed as % injected dose.

^c^ Femur:blood ratio.

*p*<0.05 vs. control (no pretreatment).

*p*<0.01 vs. control (no pretreatment).

**Table 7:** Biodistribution of radioactivity after intravenous administration of ^67^Ga-DOTA-Bn-SCN-HBP in mice with pretreatment of alendronate (20 mg/kg).^a^

	Time after administration		
Tissue	10 min	1 h	3 h
Blood	2.36 (0.20)**	0.87 (0.10)**	0.26 (0.05)**
Liver	1.59 (0.28)**	1.16 (0.21)**	0.54 (0.19)**
Kidney	13.84 (2.67)*	8.13 (1.39)**	6.42 (1.27)**
Small-intestine	0.61 (0.08)*	0.55 (0.07)**	0.38 (0.12)**
Large-intestine	0.37 (0.05)*	0.18 (0.01)**	0.38 (0.11)**
Spleen	1.42 (0.19)**	1.10 (0.25)**	0.69 (0.36)**
Pancreas	0.87 (0.15)**	0.36 (0.09)**	0.25 (0.06)**
Lung	3.06 (0.78)**	1.78 (0.18)**	0.57 (0.22) **
Heart	0.97 (0.22)*	0.43 (0.05)**	0.17 (0.05)*
Stomach^b^	0.47 (0.06)**	0.33 (0.05)**	0.23 (0.10)**
Bone (Femur)	8.59 (0.81)**	13.47 (1.76)**	13.30 (2.10)**
Muscle	0.57 (0.10)	0.35 (0.14)	0.19 (0.01)
Brain	0.06 (0.02)	0.04 (0.01)	0.02 (0.01)**
F/B ratio^c^	3.64 (0.20)**	15.82 (3.48)**	51.38 (5.91)**

^a^ Expressed as % injected dose. Each value represents the mean (SD) for five or six animals.

^b^ Expressed as % injected dose.

^c^ Femur:blood ratio.

*p*<0.05 vs. control (no pretreatment, Reference 29).

*p*<0.01 vs. control (no pretreatment, Reference 29).

### Protein-binding Assay

**Figure 5:**
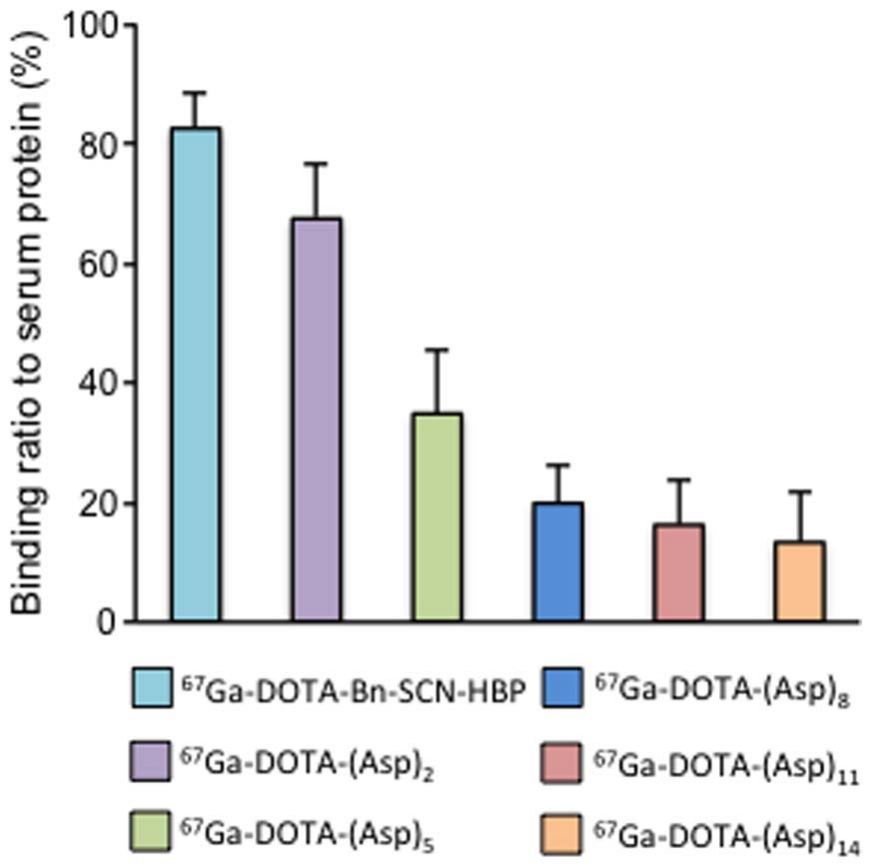
Serum protein binding. Serum protein binding ratios of ^67^Ga-DOTA-(Asp)_n_ (n = 2, 5, 8, 11, or 14) and ^67^Ga-DOTA-Bn-SCN-HBP. Data are expressed as the mean ± SD for four samples.

Proportions of ^67^Ga-DOTA-(Asp)_n_ (n = 2, 5, 8, 11, or 14) and ^67^Ga-DOTA-Bn-SCN-HBP bound to serum protein (Figure 5) shows that the binding of ^67^Ga-DOTA-(Asp)_n_ compounds to serum proteins decreased with an increase in the length of the aspartic acid chain. The serum protein binding rate of ^67^Ga-DOTA-Bn-SCN-HBP was greater than that of ^67^Ga-DOTA-(Asp)_n_ compounds.

## Discussion

**Figure 6:**
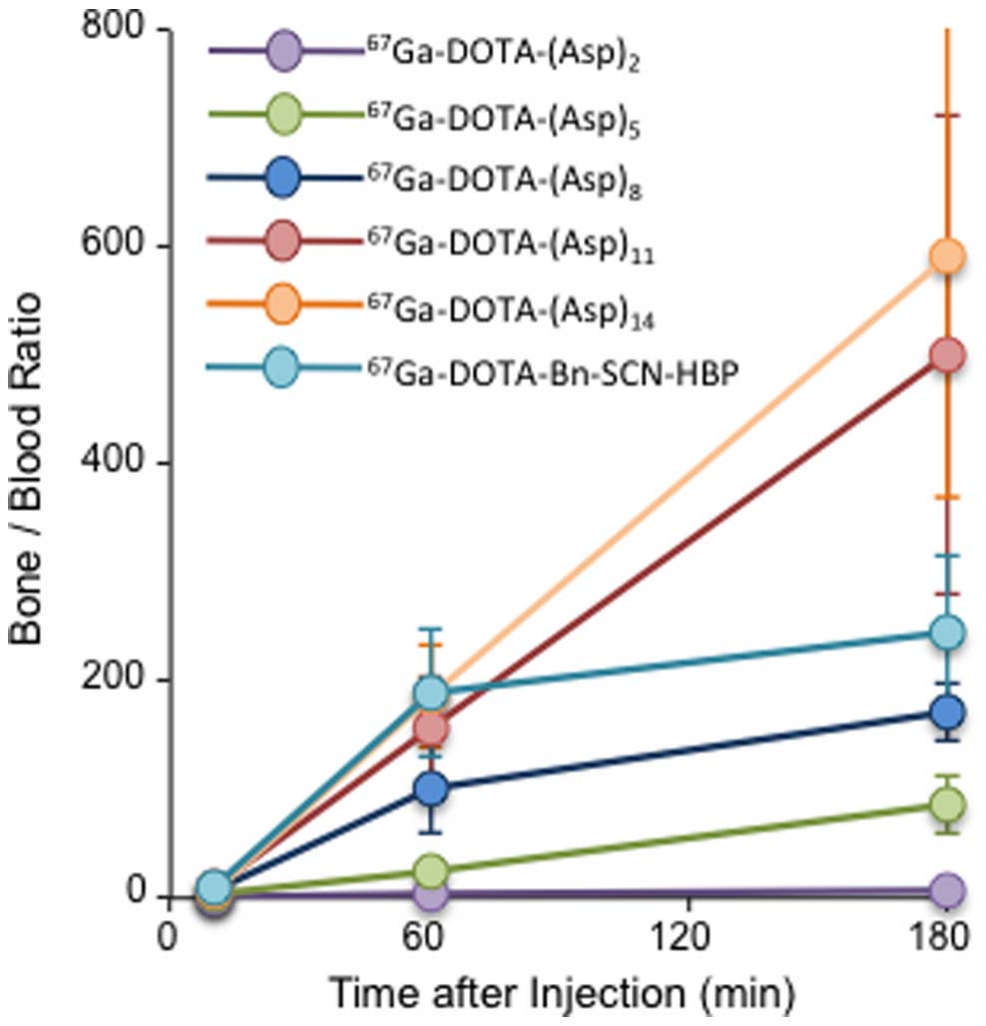
Bone/blood radio. Bone/blood ratio of radioactivity after injection of ^67^Ga-DOTA-(Asp)_n_ (n = 2, 5, 8, 11, or 14) and ^67^Ga-DOTA-Bn-SCN-HBP. Data are expressed as the mean ± SD.

Previously, we introduced the concept of radiometal complex-conjugated bisphosphonate compounds for the development of bone-seeking radiopharmaceuticals [42], [43]. Moreover, in recent years, superior activities of newly developed radiogallium complex-conjugated bisphosphonate compounds have been reported by us and other groups [30]–[34]. In these drug compounds, the bisphosphonate structure has high affinity for hydroxyapatite, which is a specific component of bone tissues, leading to targeting of bone tissues. In a previous study, it was reported that the *in vitro* binding profile of Fmoc-(Asp)_n_ (n = 2, 4, 6, 8, or 10) to hydroxyapatite increased with the increase in the length of the peptide [44]. Here a similar strategy was applied using aspartic acid peptides as the carrier to bone tissues instead of bisphosphonate. Indeed, we have demonstrated that the binding of ^67^Ga-DOTA-(Asp)_n_ (n = 5, 8, 11, or 14) to hydroxyapatite beads increased with increased length of the aspartic acid peptide. This result is consistent with the previous study and was reflected by bone accumulation of ^67^Ga-DOTA-(Asp)_n_ in biodistribution experiments. Moreover, these biodistribution experiments showed greater bone accumulation with increasing length of the peptide conjugates from ^67^Ga-DOTA-(Asp)_2_ to ^67^Ga-DOTA-(Asp)_11_. The longer compounds ^67^Ga-DOTA-(Asp)_11_ and ^67^Ga-DOTA-(Asp)_14_ accumulated equally in bone and showed superior biodistribution characteristics as that of bone imaging radiopharmaceuticals, with high accumulation in bone and rapid clearance from other tissues. Despite lower bone accumulation than the bisphosphonate ^67^Ga-DOTA-Bz-SCN-HBP, the bone/blood ratios of radioactivity after injection of ^67^Ga-DOTA-(Asp)_11_ and ^67^Ga-DOTA-(Asp)_14_, which are an index as bone imaging, were comparable or higher (Figure 6), presumably due to more rapid blood clearance than ^67^Ga-DOTA-Bz-SCN-HBP. This may reflect the lower serum protein binding ratios of ^67^Ga-DOTA-(Asp)_11_ and ^67^Ga-DOTA-(Asp)_14_ compared to that of ^67^Ga-DOTA-Bn-SCN-HBP (Figure 5).

We assumed that the high accumulation of radioactivity in the bone after injection of these compounds was due to hydroxyapatite binding of bisphosphonate or aspartic acid structures in bone tissues. To estimate the hydroxyapatite binding of these compounds, alendronate inhibition experiments were performed *in vitro* and *in vivo*. In these hydroxyapatite binding assays, ^67^Ga-DOTA-(Asp)_14_ and ^67^Ga-DOTA-Bn-SCN-HBP binding was inhibited by alendronate, confirming that the mechanism by which ^67^Ga-DOTA-(Asp)_14_ and ^67^Ga-DOTA-Bn-SCN-HBP accumulate in bone involves coordination of their functional groups to the Ca^2+^ in hydroxyapatite crystals [45]. However, ^67^Ga-DOTA-Bn-SCN-HBP binding was inhibited by lower concentrations of alendronate compared with ^67^Ga-DOTA-(Asp)_14_. Moreover, in biodistribution experiments, the inhibition of radioactive bone accumulation by alendronate was greater after injection of ^67^Ga-DOTA-Bn-SCN-HBP than that of ^67^Ga-DOTA-(Asp)_14_. Although the precise mechanisms remain unclear, the binding patterns of these compounds to hydroxyapatite may differ. Wang *et al*. reported that alendronate and (D-Asp)_8_, which were used as bone-targeting moieties on conjugated fluorescein isothiocyanate (FITC)-labeled *N*-(2-hydroxypropyl)methacrylamide (HPMA) copolymers (P-ALN-FITC and P-D-Asp_8_-FITC). In the study, P-D-Asp_8_-FITC preferentially bound bone resorption surfaces, whereas P-ALN-FITC appeared to bind both formation and resorption surfaces in bone [46]. In hydroxyapatite binding experiments with different crystallinity, P-D-Asp_8_-FITC showed preferential binding to hydroxyapatite of higher crystallinity compared with P-ALN-FITC. These observations indicated that bisphosphonate and aspartic acid peptides have different modes of hydroxyapatite binding. Accordingly, ^68^Ga-DOTA-(Asp)_14_ PET imaging may give different information than that obtained by ^99m^Tc-MDP bone scintigraphy methods. Since ^99m^Tc-MDP mainly accumulates osteoblastic lesions in bone, it has been known that sensitivity ^99m^Tc-MDP often shows false-negative in osteolytic bone metastases lesions, and consequently, its sensitivity is reduced [47]. On the contrary, ^68^Ga-DOTA-(Asp)_14_ PET imaging may have a potential to improve its sensitivity. Meanwhile, in a previous ^99m^Tc-MDP-bone scintigraphy study, treatments with bisphosphonate and alendronate may have caused false negative scintigraphy by producing competition between the drug and tracer, and blocking entrapment and accumulation of the tracer in bone [48]. In this study, pretreatment with alendronate inhibited bone accumulation of ^67^Ga-DOTA-Bn-SCN-HBP more effectively than that of ^67^Ga-DOTA-(Asp)_14_, suggesting that bisphosphonate-induced false negative scintigraphy is less likely to occur in ^68^Ga-DOTA-(Asp)_14_ PET.

Radiogallium complexes of 1,4,7-triazacyclononane-triacetic acid (NOTA) or triazacyclononane-phosphinate (TRAP) may produce radiocomplexes with a higher specific activity than DOTA, allowing the use of much lower concentrations of precursor for labeling [49]. Despite this, DOTA was used in this study because unlike receptor imaging, much higher specific activity is not necessary for hydroxyapatite-targeted bone imaging. Moreover, DOTA is more versatile and could be developed for both imaging and therapeutics. Since the DOTA ligand forms a stable complex with not only gallium (^67/68^Ga), but also lutetium (^177^Lu) and yttrium (^90^Y), which are beta particle emitters as radionuclides for therapy, furthermore, bismuth (^213^Bi) as an alpha emitter could be applicable, its application to therapy from diagnosis could be made available. That is, radiometal complexes of DOTA-(Asp)_n_ for radionuclide therapy could be useful as agents for the palliation of metastatic bone pain.

In conclusion, the ^67^Ga-DOTA complex-conjugated aspartic acid peptides^ 67^Ga-DOTA-(Asp)_n_ showed ideal biodistribution characteristics as bone scintigraphy agents. Therefore, these agents may facilitate the drug design of PET tracers with ^68^Ga for the diagnosis of bone disorders, such as bone metastases. Further studies are required to determine whether ^68^Ga-DOTA-(Asp)_n_ can provide additional information to that of bone scintigraphy, and to develop these compounds for radionuclide therapy.
